# Long-Term Follow-Up After Surgical Treatment of Anogenital and Oropharyngeal Condyloma Acuminata with Simultaneous HPV Vaccination Initiation in Patients with Concurrent HPV-16 or -18 Co-Infection

**DOI:** 10.3390/jcm15187005

**Published:** 2026-09-10

**Authors:** Joanna Wojciula, Marcin Bartoszewicz, Magdalena Wojciula, Piotr Sienkiewicz, Grzegorz Szewczyk, Piotr Fiedor

**Affiliations:** 1 Department of Obstetrics, Gynaecology and Gynaecologic Oncology, St. John Paul II Mazovian Regional Hospital in Siedlce, Poniatowskiego Street 26, 08-110 Siedlce, Polandgrzegorz.szewczyk@wum.edu.pl (G.S.); 2 Warsaw Medical Innovation Center JSC, Béli Bartóka 8/25, 02-787 Warsaw, Poland; 3 Medical University of Warsaw, Żwirki i Wigury 61, 02-091 Warsaw, Poland; 4 Faculty of Medical and Health Sciences, University of Siedlce, Konarskiego 2, 08-110 Siedlce, Poland; 5 Medical University of Bialystok, Jana Kilińskiego 1, 15-089 Białystok, Poland; 6 Department of General and Vascular Surgery, St. John Paul II Mazovian Regional Hospital in Siedlce, Poniatowskiego Street 26, 08-110 Siedlce, Poland; 7 Institute of Health Sciences, University of Siedlce, Konarskiego 2, 08-110 Siedlce, Poland; 8 Department of Biophysics, Physiology and Pathophysiology, Medical University of Warsaw, Żwirki i Wigury 61, 02-091 Warsaw, Poland; 9 VIZJA University, Okopowa 59, 01-043 Warsaw, Poland

**Keywords:** peri-operative vaccination, human papillomavirus, condyloma acuminata, anogenital, oropharyngeal

## Abstract

**Background/Objectives**: Persistent infection with high-risk HPV genotypes accounts for the development of a substantial portion of anogenital and oropharyngeal malignancies. Patients with primary and secondary immunodeficiency or those receiving immunosuppressive therapy, including transplant recipients and patients affected by rare diseases, are vulnerable to particularly severe clinical manifestations of infection. While HPV vaccination constitutes primary prevention, patients with HPV-associated disease remain at risk of reinfection or subsequent infection with alternative genotypes following surgical treatment and may benefit from peri-operative vaccination regimens. This study aimed to evaluate long-term outcomes of HPV vaccination following surgical management of condyloma acuminata in patients with concurrent high-risk genotype infection. **Methods**: 350 patients with condyloma acuminata and HPV-16 or -18 co-infection confirmed by histopathological examination and PCR genotyping (oral cavity, vulva, vagina, penis, anoderma), including a subgroup with primary or acquired immunodeficiency or chronic immunosuppressive regimens, underwent surgical treatment, receiving simultaneous immunization with the first dose (subsequent completion of full regimen over 12 months) of the bivalent Cervarix vaccine (170 patients) or the quadrivalent Gardasil vaccine (180 patients). A follow-up examination, including HPV genotyping (16/18/31/35), was performed after 6 and 12 months following regimen completion. After 36 months, a standard clinical examination was performed, and in doubtful cases, supplemented with genotyping and biopsy/cytology. Observation was continued in subsequent years (5, 10, and >15 years). **Results**: Condyloma acuminata requiring removal were observed in 5% of the patients in follow-up at 36 months, with no significant statistical difference between vaccine groups. No cellular atypia or malignant transformation in the form of squamous cell carcinoma was observed. Genotyping did not detect HPV-16/18/31/35 infection in the follow-up examinations. **Conclusions**: In this uncontrolled, single-arm cohort, peri-operative initiation of either the bivalent or quadrivalent HPV vaccine was associated with a low rate of clinically apparent condyloma acuminata recurrence and no detectable HPV-16/18/31/35 infection during follow-up.

## 1. Introduction

Human papillomavirus (HPV) infection results in a wide array of clinical manifestations, impacting a variety of anatomical sites. Condyloma acuminata are associated with low-risk HPV (LR-HPV) genotypes 6 and 11. These benign lesions of varying appearance can occur at any site of sexual contact, with the virus infecting mucosal or cutaneous epithelium, affecting the penis, vulva, vagina, cervix, anoderma, or oropharynx, or the surrounding skin [1].

HPV genotypes 16 and 18 have the highest oncogenic potential and are the most commonly detected subtypes in cases of cervical and anal cancers, and of their precursor lesions, globally. High-risk HPV (HR-HPV) genotypes, comprising genotypes 16, 18, 31, 33, 35, 39, 45, 51, 52, 56, 58, 59, 68, 73, and 82, are also associated with malignancies of the vulva (70%), vagina (75%), head and neck (26–30%) including oropharynx (30%), and penis (60% of penile cancers, 79.8% of penile intraepithelial neoplasia) [2].

While anal cancer is rare in the general population, certain populations are significantly impacted, including transgender people assigned male at birth (TG-AMAB) and men who have sex with men (MSM). A 2024 study found HR-HPV prevalence in a TG-AMAB cohort to be comparable to literature values reported for MSM. Among individuals with HIV, HR-HPV prevalence was 80% in the TG-AMAB cohort compared to 74% reported in MSM. Among HIV-negative individuals, the values were 62% and 41%, respectively. The prevalence figures are substantially higher than those reported in the literature for men who have sex with women (27% HIV+, 7% HIV−) [3].

While 90% of HPV infections are estimated to clear naturally within two years of detection, persistent infection may progress to cytological abnormality. This supports vaccination efforts, with evidence from pre- and post-licensure clinical trials and studies supporting vaccine safety and efficacy [4].

HPV vaccination programs led to significant population-level declines in incidence of cervical and vaginal precancerous and cancerous lesions, as well as of condyloma acuminata among young women [5]. A meta-analysis showed significant and substantial impact of HPV vaccination on HPV-16 and 18 infections, condyloma acuminata diagnoses, and high-grade squamous intraepithelial lesions (HSIL) among girls and women [6].

Similar trends have been observed in oropharyngeal and anal infection rate reduction, translating to prevention of subsequent malignancy development. Systematic review studies have shown an 82.7% relative risk reduction for oropharyngeal infections after vaccination and a 58% relative risk reduction in anal cancer incidence after vaccination compared to unvaccinated control groups [7].

Currently five HPV vaccines are WHO-prequalified: three bivalent (genotypes 16 and 18), including the Cervarix vaccine (in a one- and two-dose regimen), one quadrivalent (genotypes 6, 11, 16, and 18)–the Gardasil vaccine (in a one-dose regimen), and one nonavalent (genotypes 6, 11, 16, 18, 31, 33, 45, 52, and 58) vaccine [8].

Both the Cervarix bivalent vaccine and the Gardasil quadrivalent vaccine have a long history of use, high immunogenicity (seroconversion rates of nearly 100%), and a track record of safety and efficacy–demonstrating prophylactic efficacy against a range of HPV-related endpoints, and some evidence for cross-protection incidence for closely related genotypes [9]. Cross-protection has been shown to be inconsistent across non-vaccine HPV types, with data suggesting effects for 31 and 45 for the bivalent and 31 for the quadrivalent vaccine, differing between studies and with wide confidence intervals. While one trial showed moderate cross-protection against HPV types 6- and 11-related persistent infection from the bivalent vaccine, several subsequent real-world studies found no such protective effect [10].

Randomized trials in women have demonstrated strong prophylactic vaccine efficacy against persistent cervical HPV-16 and -18 infection and associated cervical intraepithelial neoplasia (CIN) grade 2 or worse lesions. The Costa Rica Vaccine Trial found this protection extended to anal and oral infection as well, with multisite efficacy highest among women naïve to HPV-16 and -18 at vaccination (83.5%), and a lower but still statistically significant multisite efficacy (57.8%) among women with serologic evidence of prior HPV-16 and -18 exposure. However, no significant vaccine efficacy against anal or oral infection was reported among women with current cervical infection at vaccination [11].

While HPV vaccines have been primarily studied and used in primary prevention, patients with clinically apparent HPV-associated disease, including condyloma acuminata, remain at risk of reinfection or subsequent infection with alternative genotypes following management. Vaccination should be recommended to reduce oncogenic risk [12].

This study aimed to evaluate long-term outcomes of HPV vaccination following surgical management of condyloma acuminata in patients with HR-HPV (16 or 18) genotype co-infection.

## 2. Materials and Methods

This retrospective, single-center, single-arm observational cohort study included 350 patients (age range: 16–62) with clinical diagnosis of condyloma acuminata in the area of the oral cavity, vulva, vagina, penis, or anoderma, and simultaneous HPV-16 or -18 co-infection confirmed by histopathological examination and PCR genotyping, who underwent surgical treatment with peri-operative vaccine initiation. Patients were eligible for inclusion if they met these clinical, virological, and treatment criteria. No exclusion criteria were established. The patient population included a subgroup of patients with primary (PID) or acquired immunodeficiency (AID), including patients undergoing chemotherapy (8 patients; age range: 28–42), or subject to chronic immunosuppression after organ transplantation (OTx) (25 patients; age range 25–62). On the day of the surgical removal of the lesions, the patients received immunization with the first dose of the bivalent Cervarix vaccine (170 patients: 150 women and 20 men) or the quadrivalent Gardasil vaccine (180 patients: 150 women and 30 men), with subsequent completion of the full respective three-dose regimen according to each vaccine’s standard schedule (Cervarix: 0, 1, and 6 months; Gardasil: 0, 2, and 6 months). In patients who presented later than scheduled for a given dose, the interval to the next dose was extended accordingly, while ensuring that the full series was completed within 12 months. No additional booster doses were administered. The follow-up examination included HPV genotyping (genotypes 16, 18, 31, and 35) and was performed after 6, 12, and 36 months from the last vaccination. The 36-month follow-up included a standard clinical examination and, in doubtful cases, biopsy/cytology.

Clinical observation was continued in subsequent years (5, 10, and >15 years). Management shifted primarily to patient-initiated follow-up, with clinical evaluations performed on appearance of clinically relevant concerns or symptoms. This shift to symptom-triggered follow-up beyond the 36-month visit means that asymptomatic recurrence or subclinical viral persistence occurring afterward may not have been captured. For the purposes of this study, recurrence was defined as the clinical appearance of new condyloma acuminata lesions following complete viral clearance, while recrudescence/relapse was defined as reappearance of lesions at or adjacent to the original treatment site, potentially reflecting incomplete excision or persistence of latent infection. As both entities were managed identically and could not be distinguished, they are reported jointly as “recurrence/recrudescence/relapse” throughout this manuscript.

Partners of patients included in the study were independently vaccinated. Partners did not show clinical signs of infection, and HPV genotyping was not performed.

The surgical procedures and vaccinations were not carried out with the aim of conducting a head-to-head study. Adverse events were not systematically graded or documented beyond the absence of patient-reported serious events. Recurrence rates between the Cervarix and Gardasil vaccine groups were compared using the chi-square test with Yates’ continuity correction for 2 × 2 tables and Fisher’s exact test [13]. As this was a retrospective, non-randomized study, no sample-size or power calculation was performed in advance.

## 3. Results

After 36 months, 100 patients in the Cervarix vaccine group and 120 patients in the Gardasil vaccine group remained under observation. After 5 years, the number of patients under observation was 60 and 40, respectively. At 10 and >15 years, a total of 30 patients remained under periodic observation. The main reasons for the reduction in cohort size over time were transfers to other centers local to the patient’s place of residence. Patients lost to follow-up were not formally compared with those retained. The possibility of attrition bias affecting the reported recurrence rate cannot therefore be excluded.

**Figure 1:**
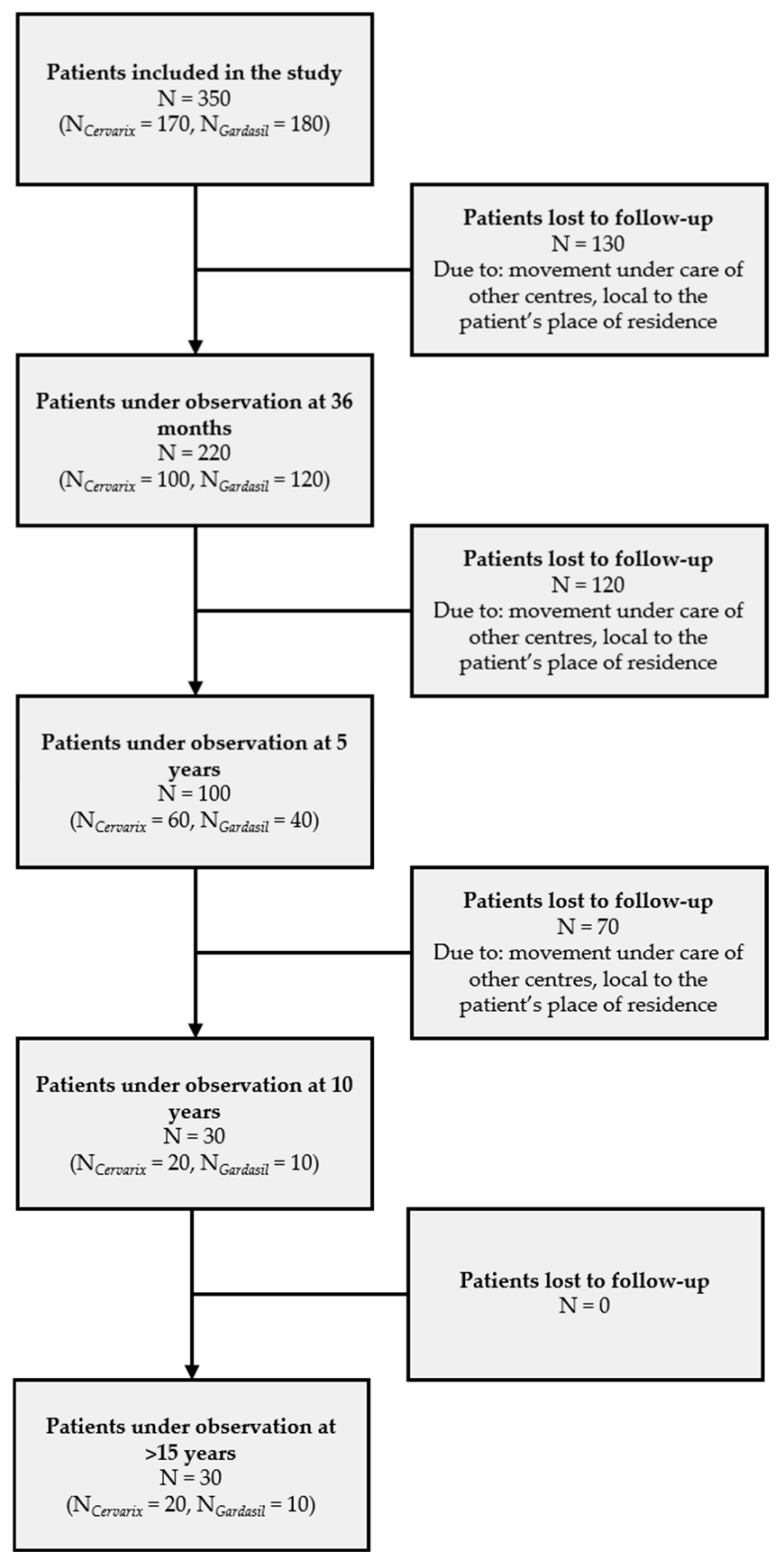
Patient flow diagram.

Figure 1 below depicts the patient flow.

Two of the patients included in the study are presented below.

**Figure 2:**
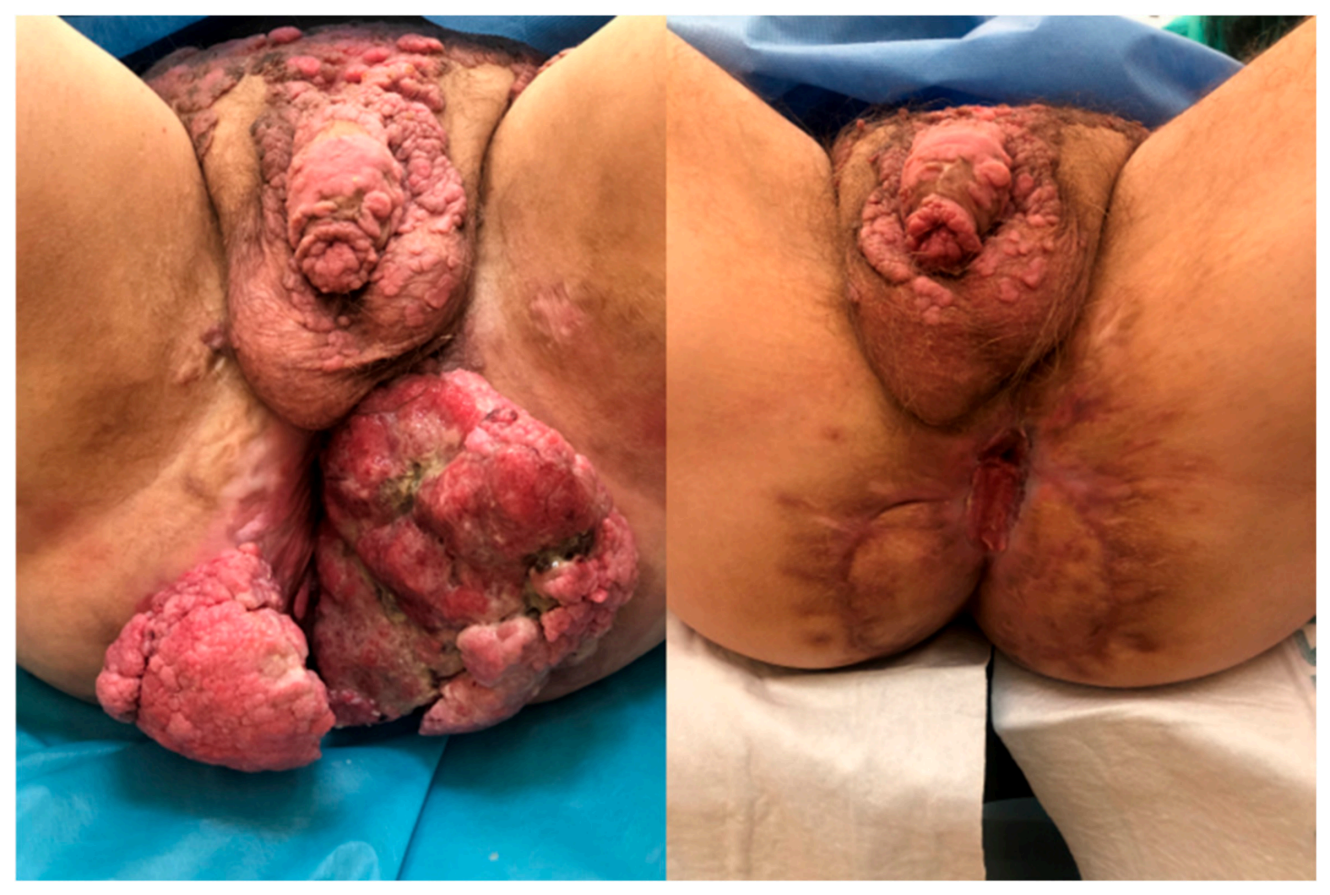
Severe clinical manifestation of anogenital condyloma acuminata and surgical treatment in a patient with PID.

Figure 2 presents severe clinical manifestations of condyloma acuminata and SCC in a PID patient, positive for HPV genotypes 6, 11, 16, and 18, prior to and during the initial surgical treatment.

**Figure 3:**
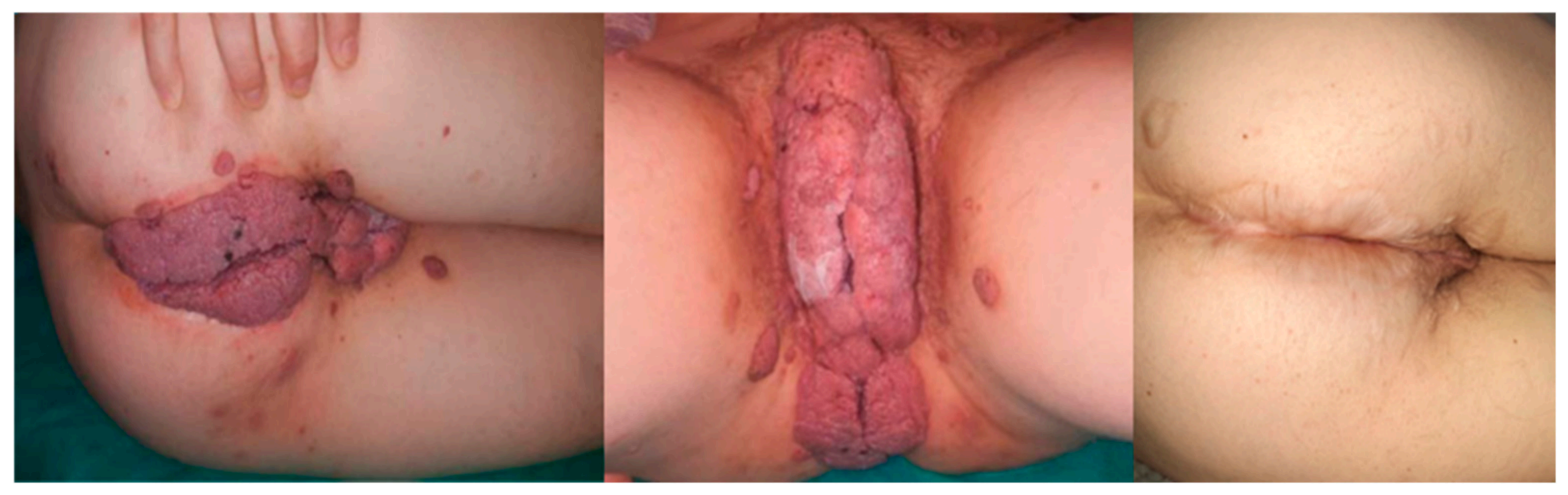
Severe clinical manifestation of anogenital condyloma acuminata and surgical treatment in a patient after kidney OTx, undergoing immunosuppression.

Figure 3 presents severe clinical manifestation of condyloma acuminata, anal intraepithelial neoplasia (AIN 3), and vulvar intraepithelial neoplasia (VIN 3) in a patient after kidney OTx, on long-term immunosuppression, prior to [14], and after surgical treatment. Control of HPV infection required adjusting immunosuppressive medication, including a switch from brand-name to generic drugs.

### 3.1. Clinical Outcomes

Up to the 36-month mark, 5% of the patients (11 out of 220 patients, 6 in the Cervarix bivalent vaccine group and 5 in the Gardasil quadrivalent vaccine group) had developed a clinical picture of condyloma acuminata requiring removal. The chi-square test with Yates’ continuity correction for 2 × 2 tables (χ^2^ = 0.10, df = 1, *p* = 0.756) and Fisher’s exact test gave a consistent result (*p* = 0.552). The difference was not statistically significant. The odds ratio was 1.47 (95% CI 0.43–4.96). Because the study was not designed or powered as a head-to-head comparison between vaccines, this finding should be interpreted descriptively rather than as evidence of comparative efficacy or equivalence.

The most frequent recurrence or recrudescence/relapse was observed in the anogenital area. The 11 patients with observed recurrence or recrudescence/relapse were patients undergoing chronic immunosuppression after OTx (kidney, liver) or undergoing planned chemotherapy due to cancer.

Table 1 presents a summary of enrolment, follow-up, and recurrence by vaccine group and by immune status.

### 3.2. Virological Outcomes

Repeated genotyping on follow-up did not detect HPV-16, -18, -31, or -35 infection, and no cases of cellular atypia or malignant transformation in the form of squamous cell carcinoma (SCC) were observed during the follow-up examinations. No serious adverse events of either vaccine were reported by the patients in the follow-up period.

## 4. Discussion

Clinicians surveyed on ideal HPV vaccination recommendations broadly indicated their support for vaccination of patients treated for cervical precancerous lesions (61.15–85.2%, dependent on the age group), treated for vaginal, vulvar, or anal cancer (50.3–73.2%, dependent on the age group), and treated for cervical cancer (47.7–67.8%, dependent on the age group). The most-supported timing for HPV vaccination in patients with CIN is during admission for treatment, with a minority of surveyed clinicians opting for postponement of vaccination, non-administration, or an alternative approach [15]. Population studies on HPV vaccination for primary cancer prophylaxis show a simultaneous significant reduction in the incidence of condyloma acuminata. The effect is particularly apparent for younger patients. The Australian vaccination program resulted in a 92.6% decrease in incidence in women younger than 21 years. Quadrivalent and nonavalent vaccines include the 6 and 11 genotypes. Previous studies in patients with a history of condyloma acuminata showed conflicting results on the use of HPV vaccination in combination treatment (with cryotherapy, imiquimod, intralesional Candida antigen, podophyllotoxin), delayed onset and reduction in the number of lesions during recurrence. The available literature suggests that HPV vaccination may mitigate, but not eliminate, the risk of recurrence [12].

The clinical picture of reappearance of condyloma acuminata in 5% of the studied population could be attributed to a recrudescence/relapse following incomplete surgical excision at the margin or to a true recurrence.

In a previous study, OTx patients had a higher morbidity of HPV-related cancer in the first 5 years after symptomatic HPV infection observation compared to patients free from immunosuppressive therapy, due to more rapid onset of high-grade intraepithelial neoplasia and invasive carcinoma [16]. Our previous experience points to higher rates of recurrence of HPV-dependent lesions among patients with PID or AID, as compared to patients receiving immunosuppression after OTx [17].

Table 2 presents a brief review of current literature data on HPV and HPV vaccination in immunocompromised patients.

Our observation of patients with HPV-16 or -18 infection and accompanying condyloma acuminata in the oral cavity, vulva, vagina, penis, or anoderma showed that simultaneous surgical removal of lesions and initiation of vaccination with a bivalent or quadrivalent vaccine was associated with a favorable clinical course in patients without significant immune response disorders in both the short- and long-term observation periods (>6 months up to 15 years). While clinical observations reported in the literature, most often indicate rates of recurrence/relapse/recrudescence of up to 50% within 6-24-36 months, in the studied group only 5% of patients (11 patients, all belonging to the immunocompromised subgroup) vaccinated around the time of surgery developed a clinical picture of condyloma acuminata requiring removal. The clinical picture of condyloma acuminata requiring removal, be that recurrence or recrudescence/relapse, was observed in patients regardless of the surgical procedure performed and whether they received the bivalent or quadrivalent vaccine around the time of surgery. This is consistent with previous studies associating occurrence, remission, relapse, and cancerization of condyloma acuminata with immune response competence or disorders [22].

HPV-16 E6/E7 oncoproteins have recently been shown, in a murine tumor model, to drive upregulation of the transcription factor KLF2 in tumor-associated macrophages, increasing local secretion of IL-23, suppressing the proliferative capacity, cytotoxicity, and interferon-γ production of HPV-specific CD8+ T-lymphocytes. Neutralization of IL-23 restored HPV-specific T-cell infiltration and synergized with therapeutic vaccination [23].

Our study did not confirm that either vaccine can fully and effectively protect immunocompromised patients from recurrence/recrudescence/relapse of condyloma acuminata. Due to the small sample size in the study, further observational studies are needed. All 11 observed recurrences occurred within the immunocompromised subgroup (patients undergoing chemotherapy or chronic immunosuppression after organ transplantation), while no recurrences were observed among immunocompetent patients. The absolute numbers nonetheless remain small, and this subgroup finding should be regarded as hypothesis-generating rather than conclusive. Future studies would benefit from inclusion of in-depth investigation into the immunocompetence of patients with recurrence/recrudescence/relapse of condyloma acuminata post-removal, particularly in the case of quadrivalent vaccine administration.

On the other hand, the observed sustained absence of detectable HPV-16, -18, -31, or -35 infection and the lack of cellular atypia or malignant transformation in the form of SCC observed on the 36-month follow-up examinations, including in the immunocompromised subgroup, is consistent with recommendations and literature-reported seroconversion rates.

It should be emphasized that the medical procedures and vaccinations carried out were not aimed at conducting a head-to-head study. A direct comparison of the two vaccines, particularly assessing the effectiveness of the quadrivalent vaccine in cases of condyloma acuminata and postoperative recurrence/recrudescence/relapse of HPV-6 and -11-dependent lesions within 6–12 months, could be planned as an observation/phase IV clinical trial, with particular regard for patients with impaired immune response and chronic immunosuppression, including those after HSCT or solid OTx. Such a study would ideally also incorporate a surgically treated, unvaccinated comparison group, to permit direct assessment of the incremental benefit of peri-operative vaccination over surgical excision alone.

### Limitations

This study has several limitations that should be considered when interpreting the findings. First, this was a retrospective, uncontrolled, single-arm cohort without randomization or an unvaccinated comparison group. Consequently, the observed low recurrence rate cannot be causally attributed to vaccination, and may equally reflect the effect of surgical clearance, patient selection, or the natural history of condyloma acuminata. The study population was also heterogeneous with respect to immune status, encompassing patients with PID, AID, OTx recipients, and immunocompetent individuals, without formal adjustment for this or other potential confounders, including smoking status, female sex, and concomitant *Ureaplasma parvum* infection, which have all been previously demonstrated to be statistically significant risk factors associated with viral persistence of genital HPV infection [24]. Other potential confounders include HIV infection, sexual behavior, number of sexual partners, lesion burden, anatomical location, and prior HPV-related disease.

Vaccine allocation was not randomized, and the study was not designed or powered as a head-to-head comparison between the bivalent and quadrivalent vaccines. The absence of a statistically significant difference between groups should not be interpreted as evidence of equivalence. Follow-up beyond 36 months relied on patient-initiated, symptom-triggered evaluation, which may have underestimated asymptomatic recurrence or subclinical viral persistence, and a substantial proportion of the initial cohort was lost to structured follow-up over time without systematic comparison between those retained and those lost. HPV antibody seroconversion was not assessed, which limits what can be said about the immune response to vaccination in this population, particularly among immunosuppressed patients. Adverse events were not systematically graded or documented beyond the absence of reported serious events. Finally, this study evaluated the bivalent and quadrivalent vaccines. As the nonavalent vaccine has since become the standard of care in many healthcare systems, the generalizability of these findings to current vaccination practice may be limited.

## 5. Conclusions

The prophylactic efficacy of both the bivalent Cervarix and quadrivalent Gardasil vaccines is well established in randomized trials. Our observations extend this evidence by supporting the safety and feasibility of initiating vaccination immediately after surgical treatment, with a low rate of clinically apparent condyloma acuminata recurrence during follow-up.

Future studies could expand on the precise timing of initiation of the vaccination regimen in the peri-operative period, assessing whether a delay or initiation of vaccination prior to treatment would affect long-term outcomes.

The immunocompromised subgroup follow-up suggests the need for development and/or broader implementation into clinical practice of standards for patients with PID and AID (transplant recipients, patients undergoing chemotherapy or dialysis procedures, or awaiting such interventions), with focus on indications and precise timing of HPV vaccination regimen initiation.

Based on the existing literature, and consistent with the low recurrence observed in the present cohort, vaccination against oncogenic HPV genotypes prior to a planned organ transplant may be a valuable strategy to reduce the subsequent development, recurrence/recrudescence/relapse of condyloma acuminata and HPV-16/18 activity in patients with weakened immunity, although this specific pre-transplant timing was not directly evaluated by the present study design. Adjusting or changing the immunosuppression regimen is an important factor in lowering cancer risk.

In this uncontrolled observational cohort, initiation of vaccination regimen immediately following treatment for condyloma acuminata in patients simultaneously positive for high-risk HPV-16 or -18 genotypes appears to be associated with low overall recurrence of condyloma acuminata and sustained absence of detectable HPV-16 and 18 infection during the long-term follow-up period.

## Figures and Tables

**Table 1:** Summary of enrolment, follow-up, and recurrence by vaccine group and by immune status.

	Cervarix (Bivalent)	Gardasil (Quadrivalent)	Total
Initial inclusion, n (F/M)	170 (150/20)	180 (150/30)	350
Under observation at 36 months, n	100	120	220
Under observation at 5 years, n	60	40	100
Under observation at 10 years, n	20	10	30
Under observation at >15 years, n	20	10	30
Condyloma acuminata recurrence by 36 months, n (%)	6 (6.0%)	5 (4.2%)	11 (5.0%)
Condyloma acuminata recurrence new cases after 36 months, n	0	0	0
Recurrences in immunocompromised subgroup, n (% of that vaccine’s recurrences)	6 (100%)	5 (100%)	11 (100%)

**Table 2:** HPV vaccines and immunocompetence.

Relevant Information/Recommendations	Group	Source
There is an increased risk of HPV-related malignancy in immunocompromised patients.	Overall immunocompromised	[18]
Quadrivalent HPV vaccination (in a three-dose regimen) is recommended prior to lung OTx for all individuals between the ages of 9 and 26. HPV vaccination is safe and effective in the lung OTx group, but high daily immunosuppressive therapy hampers response.	Lung OTx candidates	[19]
Lower immune response is observed in kidney OTx recipients compared to patients with chronic kidney disease or those undergoing dialysis. HPV vaccination is preferred prior to OTx but is safe and effective both pre- and post-OTx.	Kidney OTx candidates/recipients	
There is an increased risk of HPV-associated cancers of the anogenital area and oropharynx after liver OTx. A three-dose regimen of HPV vaccination is recommended.	Liver OTx candidates/recipients	
Most studies in immunocompromised patients involve the use of the quadrivalent HPV vaccine (in a standard three-dose regimen). Immune responses are robust but attenuated in older patients and patients receiving immunosuppressive therapy.	Overall immunocompromised	[20]
Routine revaccination (HPV) is recommended post-HSCT. HPV vaccination is safe and effective, but lower immunogenicity is noted for patients receiving immunosuppressive therapy or rituximab.	HSCT recipients	
HPV vaccination results in high seroconversion rates, comparable to healthy controls.	Biologic treatment for inflammatory bowel diseases	
HPV vaccination is associated with high to very high rates of seropositivity for HPV-16 and -18 in cancer survivors, HSCT and solid OTx recipients, and healthy participants. Little to no difference in seropositivity rates is observed in immunocompromised groups compared to healthy participants for HPV-16 and -18 at 7 months.	Overall immunocompromised (non-HIV)	[21]
Most studies did not identify serious adverse events of HPV vaccination in the immunocompromised populations.	Overall immunocompromised (non-HIV)	

## Data Availability

The raw data supporting the conclusions of this article will be made available by the authors on request.

## Abbreviations

The following abbreviations are used in this manuscript:

AID	Acquired immunodeficiency
AIN	Anal intraepithelial neoplasia
CIN	Cervical intraepithelial neoplasia
HPV	Human papillomavirus
HR-HPV	High-risk human papillomavirus
HSCT	Hematopoietic stem cell transplantation
HSIL	High-grade squamous intraepithelial lesion
LR-HPV	Low-risk human papillomavirus
MSM	Men who have sex with men
OTx	Organ transplantation
PID	Primary immunodeficiency
SCC	Squamous cell carcinoma
TG-AMAB	Transgender people assigned male at birth
VIN	Vulvar intraepithelial neoplasia
